# Investigation of the Mechanical Properties of Calcareous Sand Improved by Polyurethane Foam Adhesive Under Fixed Principal Stress Axes Shearing

**DOI:** 10.3390/polym17050644

**Published:** 2025-02-27

**Authors:** Dan Chang, Yongjun Xie, Xinghua Zhang, Jiankun Liu

**Affiliations:** 1 School of Civil Engineering, Sun Yat-sen University, Guangzhou 510275, China; xieyj53@mail2.sysu.edu.cn (Y.X.); zhangxh263@mail2.sysu.edu.cn (X.Z.); liujiank@mail.sysu.edu.cn (J.L.); 2 Guangdong Key Laboratory of Marine Civil Engineering, School of Civil Engineering, Sun Yat-sen University, Guangzhou 510275, China; 3 State Key Laboratory for Tunnel Engineering, Guangzhou 510275, China; 4 Guangdong Research Center for Underground Space Exploitation Technology, School of Civil Engineering, Sun Yat-sen University, Guangzhou 510275, China

**Keywords:** polyurethane foam adhesive (PFA), calcareous sand improvement, non-coaxial characteristics, stress lode angle, strength criterion

## Abstract

The mechanical properties and envelope curve predictions of polyurethane-improved calcareous sand are significantly influenced by the magnitude and direction of principal stress. This study conducted a series of directional shearing tests with varying polyurethane contents (*c* = 2.5%, 5%, and 7.5%), stress Lode angles ($\theta_{\sigma }\;$ = −19.1°, 0°, 19.1°, and 30°), and major principal stress angles (*α* = 0°, 30°, 45°, 60°, and 90°) to investigate the strength and non-coaxial characteristics of calcareous sand improved by polyurethane foam adhesive (PFA). Key findings revealed that failure strength varied significantly with the major principal stress axis direction, initially decreasing to a minimum at α = 45° before increasing, with a 30% decrease and 25% increase observed at *c* = 5%. Non-coaxial characteristics between strain increment and stress directions became more pronounced, with angles varying up to 15°. Increasing polyurethane content from 2.5% to 7.5% enhanced sample strength by 20% at $\theta_{\sigma }$ = −19.1° and *α* = 60°. A generalized linear strength theory in the π-plane accurately described strength envelope variations, while a modified Lade criterion, incorporating polymer content, effectively predicted multiaxial strength characteristics with less than 10% deviation from experimental results. These contributions provide quantitative insights into failure strength and non-coaxial behavior, introduce a robust strength prediction framework, and enhance multiaxial strength prediction accuracy, advancing the understanding of polyurethane-improved calcareous sand for engineering applications.

## 1. Introduction

Calcareous sand, a granular material abundant in marine environments, is distinguished by its high CaCO_3_ content, setting it apart from terrestrial sand [1]. Its widespread availability has made it a preferred material for civil engineering projects, particularly in island and reef construction. However, its engineering application is hindered by several inherent properties, including irregular particle morphology, high porosity [2,3,4,5], significant compressibility [2,6], and notable fragility [3,7]. These characteristics restrict its direct use as a foundation material in marine engineering projects [8], necessitating reinforcement treatments to meet engineering standards. Traditional stabilization methods using lime and cement have proven effective in enhancing the engineering properties of calcareous sand foundations. However, these conventional modifiers, particularly Portland cement, calcite powder, and gypsum [9,10,11,12], raise environmental concerns due to their alkaline nature, which can adversely affect marine ecosystems [13].

Given the ecological sensitivity of marine environments [14], there is an increasing demand for environmentally sustainable reinforcement materials. Polyurethane, a high-molecular-weight polymer, has gained attention as a viable alternative due to its rapid strength development, low density, superior adhesion, excellent toughness, and long-term environmental stability [15,16,17]. Unlike traditional materials, polyurethane is non-water-sensitive, maintaining structural integrity without significant shrinkage or deterioration over time [5,18,19]. Its efficacy in soil reinforcement has been demonstrated in various geotechnical applications, such as highway and railway subgrades [20], embankments, and slope stabilization [21,22,23].

For example, Xiao et al. [24] reported significant improvements in peak and residual strength, as well as enhanced ductility, in polyurethane-treated sand. Woodward et al. [25] also documented substantial reductions in embankment settlement through polyurethane grouting in railway ballast stabilization. Liu et al. [13] further validated the reduction in residual deformation in polymer-reinforced materials through dynamic triaxial tests.

Beyond laboratory studies, numerous field applications have underscored the practical benefits of polyurethane foam adhesive (PFA) in geotechnical engineering. Wang et al. [26] recently utilized PFA to stabilize calcareous sand for coastal foundation construction, achieving a 30% increase in bearing capacity and a significant reduction in post-construction settlement. Similarly, Chen et al. [27] demonstrated that PFA-treated soil exhibited a 50% improvement in cohesion compared to untreated soil, effectively addressing slope instability during heavy rainfall in mountainous regions. In another case, Li et al. [28] successfully applied PFA to reinforce the foundation of a coastal highway in Southeast Asia, where calcareous sand is prevalent, resulting in markedly reduced settlement and enhanced bearing capacity. These findings collectively highlight the versatility and effectiveness of polyurethane-based materials in addressing geotechnical challenges while aligning with ecological sustainability goals.

Despite these advancements, the mechanical behavior of polyurethane-reinforced calcareous sand under complex stress conditions remains poorly understood. Current research primarily focuses on the evolution of strength and deformation parameters, lacking a comprehensive constitutive model that can describe its behavior under complex stress paths. Moreover, the influence of strain rate on the mechanical response of this material system has been insufficiently investigated [29], especially in scenarios involving principal stress axis rotation. This gap is particularly critical for island and reef engineering, where the anisotropy of sandy soil significantly influences strength, deformation, and dilatancy behaviors [30,31,32,33]. While several strength criteria incorporating principal stress axis rotation have been developed [34,35,36], they have not been adequately applied to reinforced calcareous sand systems.

This study aims to address this gap by investigating the strength characteristics of polyurethane-reinforced calcareous sand under complex stress states using a hollow cylinder torsional shear test apparatus. The research focuses on understanding the non-coaxial characteristics under different principal stress directions and employs the modified Lade criterion to construct experimental failure surfaces in the π-plane and three-dimensional principal stress space. Additionally, the influence of polymer content is systematically incorporated into the strength criterion, providing a more comprehensive understanding of the mechanical behavior. By doing so, this study not only advances the theoretical framework for reinforced calcareous sand but also offers practical insights for its application in environmentally sensitive marine engineering projects.

## 2. Test Conditions

### 2.1. Test Apparatus

**Figure 1:**
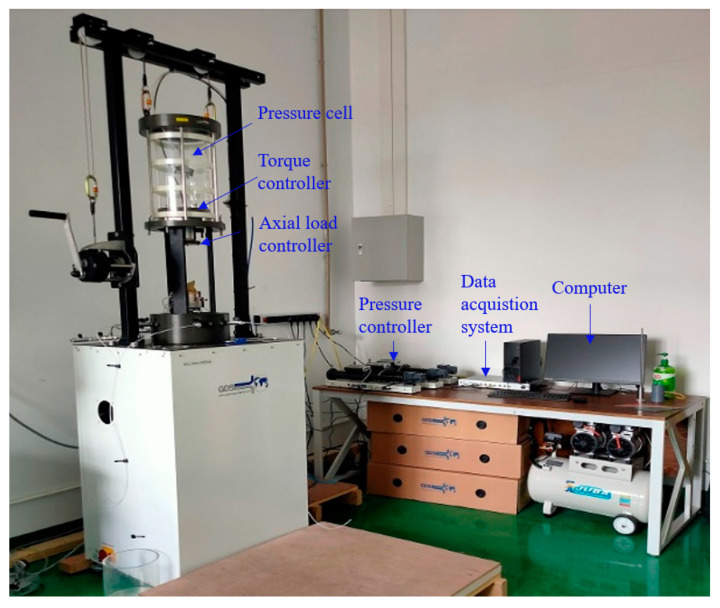
The physical setup diagram of the dynamic hollow cylinder apparatus for sandy soil materials.

The experimental investigations in this study were conducted using the SS-HCA (Figure 1) hollow cylinder torsional shear apparatus developed by Sun Yat-sen University. This advanced testing system integrates several key components: a pressure chamber, an axial–torsional loading system, independent internal and external confining pressure control systems, and a sophisticated data acquisition and processing unit. The SS-HCA apparatus offers significant advantages for geotechnical research, particularly its capability to precisely simulate constant principal stress axis rotation paths. The system’s unique design allows independent control of axial force, torque, and both internal and external confining pressures, facilitating flexible manipulation of principal stress orientation and magnitude to meet diverse requirements in civil engineering research. The technical specifications and key features of the SS-HCA apparatus are systematically presented in Table 1.

**Figure 2:**
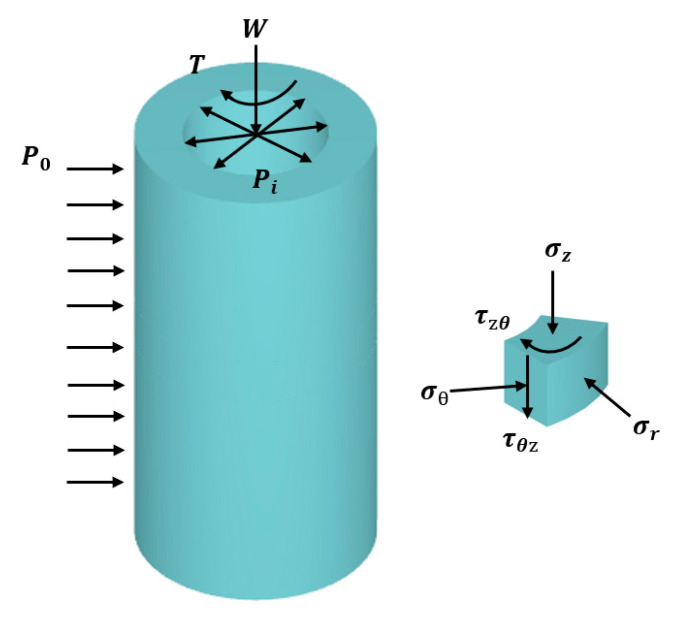
Stress state of hollow cylindrical specimen element.

Figure 2 illustrates the stress state analysis of a thin-walled element within a hollow cylindrical specimen. Following the methodology established by Hight et al. [37], the specimen’s mechanical behavior was controlled through four independent parameters: axial force (*W*), torque (*T*), internal confining pressure ($P_{i}$), and external confining pressure ($P_{0}$). This control system enables the determination of stress components, including axial stress ($\sigma_{z}$), radial stress ($\sigma_{r}$), hoop stress ($\sigma_{\theta }$), and torsional shear stress ($\tau_{z\theta }$). Subsequently, the corresponding strain components—axial strain ($\varepsilon_{z}$), radial strain ($\varepsilon_{r}$), hoop strain ($\varepsilon_{\theta \;}$), and shear strain ($\gamma_{z\theta }$)—can be derived. Furthermore, this experimental approach allows for the calculation of fundamental stress parameters: mean principal stress (*p*), deviatoric stress (*q*), intermediate principal stress coefficient (*b*), and principal stress axis rotation angle (*α*).

### 2.2. Test Materials

**Figure 3:**
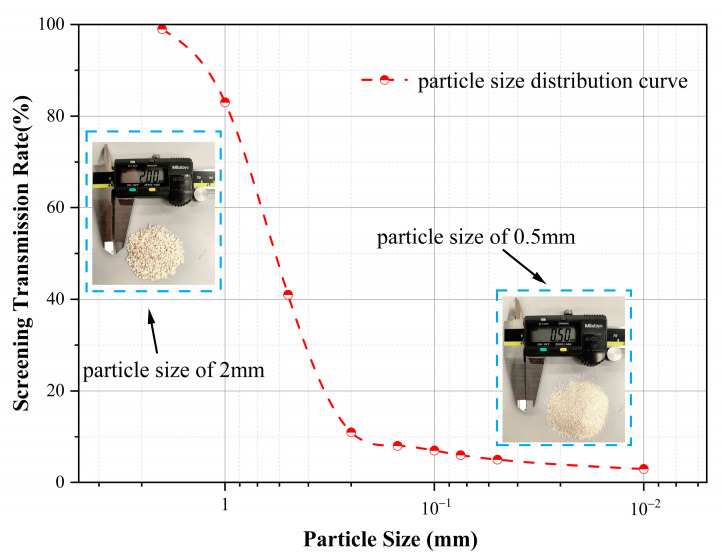
Particle size distribution curve of calcareous sand.

In this study, calcareous sand from a reef in the South China Sea was selected as the experimental material. The natural calcareous sand particles are uncemented, and their irregular shapes pose a risk of damaging the rubber membrane during testing. To ensure the accuracy and reliability of the hollow torsional shear test, only particles smaller than 2 mm were used. Prior to specimen preparation, the sand was thoroughly cleaned to remove salt residues and impurities introduced during transportation, thereby minimizing potential interference with subsequent tests. Compared to conventional quartz sand, calcareous sand generally exhibits a higher specific gravity. While the specific gravity of ordinary quartz sand typically ranges between 2.60 and 2.70, that of calcareous sand is slightly higher, ranging from 2.70 to 2.79. The particle size distribution curve of the calcareous sand is presented in Figure 3. The uniformity coefficient and curvature coefficient were determined to be 2.85 and 0.52, respectively, indicating that the sand is well-graded.

**Figure 4:**
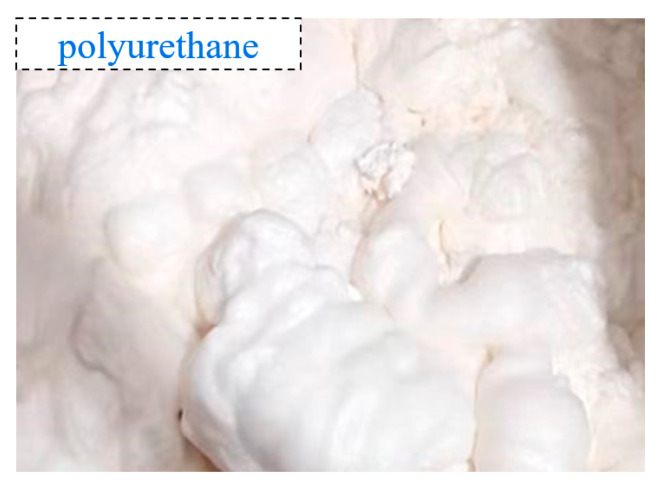
Cured polyurethane in the experiment.

In this study, a single-component polyurethane foam adhesive (Figure 4) was employed as the curing agent. The adhesive demonstrates a minimum shear strength of 80 kPa, indicative of its robust mechanical properties. The foaming process is characterized by a surface curing time of approximately 5 min, with complete curing achieved within 20 min. Following a stabilization period of 3 to 5 h, the cured foam exhibits a thermal resistance range from −30 to 80 °C, rendering it adaptable to diverse thermal environments. Polytetrafluoroethylene (PFA), the principal constituent of the curing agent, is recognized for its non-toxic and eco-friendly attributes [16,17], as well as its superior water resistance. These features ensure that the polymer-cured island reef structures retain enduring stability and strength in the variable marine setting, thereby providing effective protection for delicate marine ecosystems and reducing the long-term environmental hazards typically associated with conventional curing materials.

### 2.3. Specimen Preparation

In this experiment, a constant sample mass of 1271 g was utilized, corresponding to a dry density of 1.33 g/cm^3^. The mass ratio of polyurethane to dry calcareous sand is defined as the polyurethane dosage, denoted as *c*%. In the context of experimental research on sandy soils, various sample preparation methods are available, including the falling sand method, the underwater vibration method, the wet vibration method, and the wet tamping method. Among these, the wet tamping method is superior in achieving the desired relative density and ensuring sample uniformity. Consequently, the layered wet tamping method was employed for sample preparation in this study.

**Figure 5:**
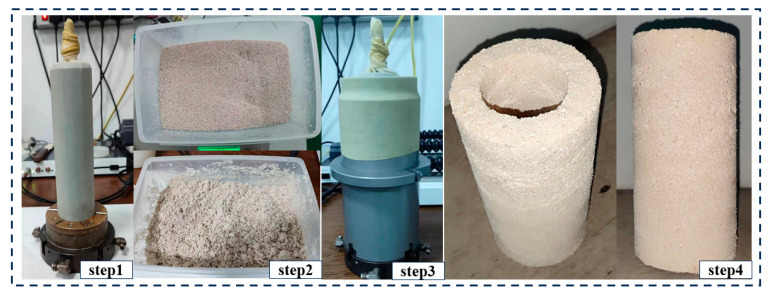
Preparation process of calcareous sand samples.

The sample preparation procedure comprises the following four steps, as illustrated in Figure 5. First, the hollow cylindrical sample preparation mold was assembled. Given the rapid solidification of consolidated calcareous sand, the mold was prepared in advance to prevent uneven consolidation during the pouring process. Second, based on the relative density specified for the experiment, the theoretical mass of the samples was calculated, thereby determining the required mass of calcareous sand. Third, after precisely weighing the calcareous sand, it was uniformly mixed into the pre-foamed polymer and rapidly stirred to ensure a homogeneous mixture. Fourth, the mixture was poured into the mold in five distinct layers, with each layer compacted sequentially. To enhance interlayer bonding, the surface of each layer was scarified before the subsequent layer was added.

The prepared specimen undergoes a saturation process to ensure optimal testing conditions. Prior to saturation, an initial confining pressure of 30 kPa is applied to stabilize the specimen and prevent potential damage during the procedure. The saturation process consists of three sequential steps. First, CO_2_ is introduced to displace the air present in the specimen and the pipeline. Next, water head saturation is conducted by injecting degassed water to replace the CO_2_. Finally, due to the large size of the hollow torsional shear specimen, backpressure saturation is employed to achieve full saturation. Once the specimen is fully saturated and consolidated, the shear test can be initiated.

### 2.4. Torsional Shear Testing Methods and Standards

The torsional shear tests were conducted using a hollow cylinder torsional shear apparatus (SS-HCA), based on the principles of directional shearing and principal stress rotation as proposed by Hight et al. [37] and Gutierrez et al. [38]. Although there is no specific international standard directly applicable to this testing apparatus, our experimental procedures strictly adhered to the general principles of standardized geotechnical testing methods. Sample preparation and compaction were carried out in accordance with ASTM D4253/D4254 [39], while the torsional shear testing procedures were adapted from ASTM D6028 [40]. The application of these standards ensures the accuracy and reliability of the experimental results.

### 2.5. The Criteria for Failure

For the failure of sandy soil under complex stress paths, researchers often adopt a specific threshold of generalized shear strain as the failure criterion. For instance, Guo et al. [41] proposed that the failure criterion for Fujian standard sand is reached when the generalized shear strain attains 5%. Similarly, Wang [42] recommended using allowable deformation as the failure criterion for soil samples. Shen [43], through an investigation of dynamic cyclic rotation in sandy soil, suggested that a generalized shear strain of 10% should be considered as the failure criterion. In this study, the specimen is deemed to have failed when the generalized shear strain reaches 6.5%.

### 2.6. Test Scheme

**Figure 6:**
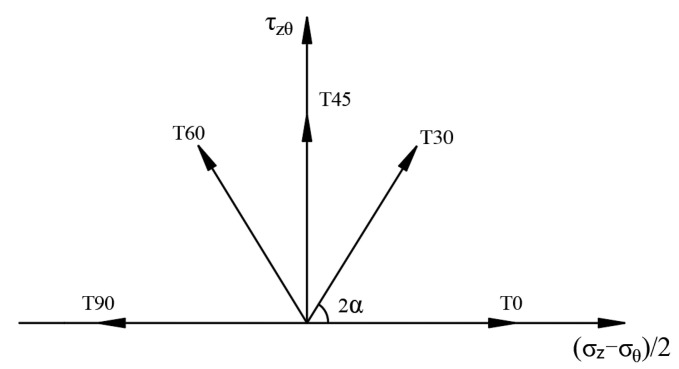
Schematic diagram of oriented shear test proposal.

To investigate the strength characteristics of solidified calcareous sand in the three-dimensional principal stress space, directional shear tests were conducted under five distinct major principal stress angles (*α* = 0°, 30°, 45°, 60°, and 90°) (Figure 6) and three polymer contents (*c* = 2.5%, 5%, and 7.5%) (Table 2). The intermediate principal stress coefficient *b* was maintained at 0.2, 0.5, 0.8, and 1. Utilizing Equations (1) and (2), the corresponding Lode angles of stress were determined as $\theta_{\sigma }$ = −19.1°, 0°, 19.1°, and 30°.

(1) $$b=\frac{\sigma_{2}-\sigma_{3}}{\sigma_{1}-\sigma_{3}}=\frac{\sqrt{3}\tan\theta_{\sigma }+1}{2}$$

(2) $$\tan\theta_{\sigma }=\frac{2\sigma_{2}-\sigma_{1}-\sigma_{3}}{\sqrt{3}\left(\sigma_{1}-\sigma_{3}\right)}=\frac{2b-1}{\sqrt{3}}$$

**Figure 7:**
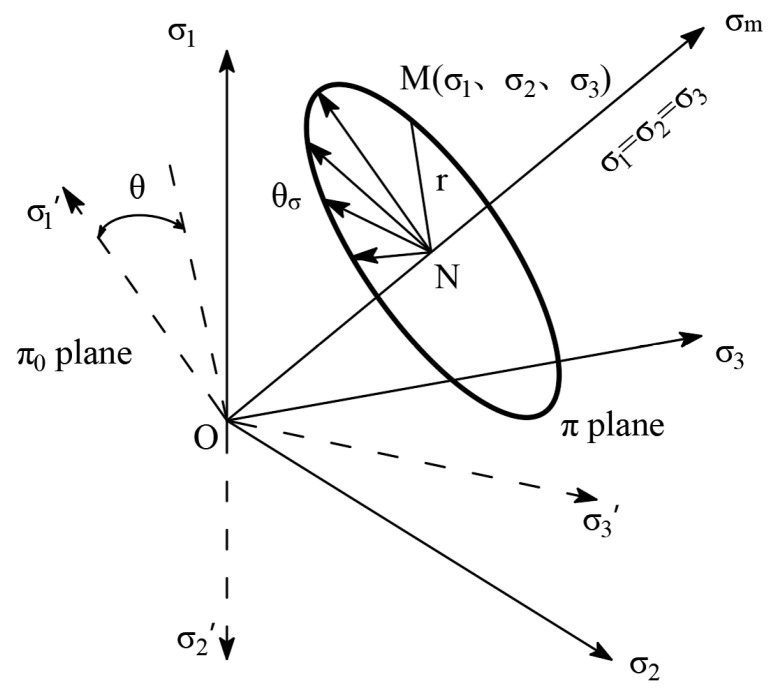
Schematic diagram of the stress path for directional shearing with different intermediate principal stress coefficients.

Figure 7 illustrates the stress paths in the π-plane for fixed-axis shear tests conducted with varying intermediate principal stress coefficients. The applied stress path consists of two stages. Initially, the specimen is loaded to attain the preset stress Lode angle. Prior to directional shearing, a 5 min period is allocated for angle stabilization and strain reading resetting. In the second stage, the direction angle of the maximum principal stress and the intermediate principal stress coefficient are held constant, while the shear stress is incrementally increased at a rate of 2 kPa/min until the generalized shear strain reaches 6.5% or the specimen fails. The intermediate principal stress coefficient b is maintained at 0.2, 0.5, 0.8, and 1.0, respectively.

## 3. Results and Analyses

The hollow cylindrical torsion and shear system sensors are designed to measure the displacement, torsion angle, and volume changes of fluids within the internal and external confining pressure controllers, as well as the backpressure controller throughout the experimental procedure. The GDSLAB v.2 module incorporates algorithms that facilitate the calculation of the specimen’s axial stress, radial stress, hoop stress, and shear stress under directional shearing conditions, as detailed in Table 3. By integrating the experimental data with Equations (3) and (4), the generalized shear stress and generalized shear strain can be accurately determined.

(3) $$q=\frac{1}{\sqrt{2}}\sqrt{{\left(\sigma_{1}-\sigma_{2}\right)}^{2}+{\left(\sigma_{1}-\sigma_{3}\right)}^{2}+{\left(\sigma_{2}-\sigma_{3}\right)}^{2}}$$

(4) $$\gamma_{g}=\frac{\sqrt{2}}{3}\sqrt{{\left(\varepsilon_{1}-\varepsilon_{2}\right)}^{2}+{\left(\varepsilon_{1}-\varepsilon_{3}\right)}^{2}+{\left(\varepsilon_{2}-\varepsilon_{3}\right)}^{2}}$$

### 3.1. Stress–Strain Relationships Under Different Major Principal Stress Angles

**Figure 8:**
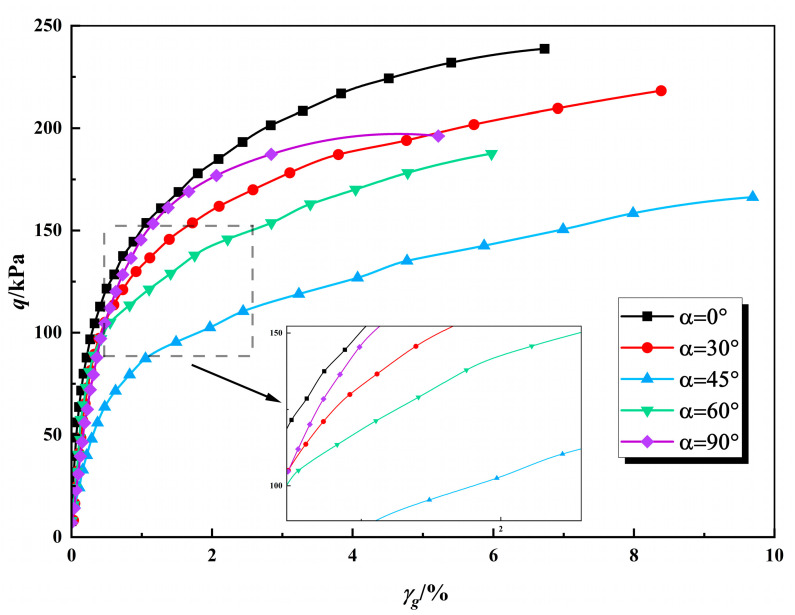
The relationship curves between generalized shear strain and deviatoric stress (*b* = 0.5, *c* = 5%).

In this study, the principal stress axis deviation angle (*α*) and the intermediate principal stress ratio (*b*) of the solidified calcareous sand specimens were kept constant throughout the shearing process, with only the deviatoric stress (*q*) being incrementally increased. Figure 8 presents the experimental relationship curves between generalized shear stress and generalized shear strain for the solidified calcareous sand specimens under drained conditions. Within the investigated range of deviation angles, the maximum shear strength was observed at *α* = 0°. As α increased from 0° to 45°, the shear strength progressively decreased from 239 kPa to 164 kPa. However, a notable increase in shear strength was observed when α varied from 45° to 90°. The degree of strain hardening was also found to be influenced by the principal stress deflection angle. Specifically, the shear strength at α = 45° was approximately 30% lower than that at *α* = 0°, demonstrating the significant impact of anisotropy on the mechanical properties. In practical island and reef engineering applications, it is crucial to account for the strength reduction caused by the deflection of the principal stress axis in structures subjected to wave and wind loads when utilizing polyurethane-solidified calcareous sand, thereby ensuring structural safety and integrity.

**Figure 9:**
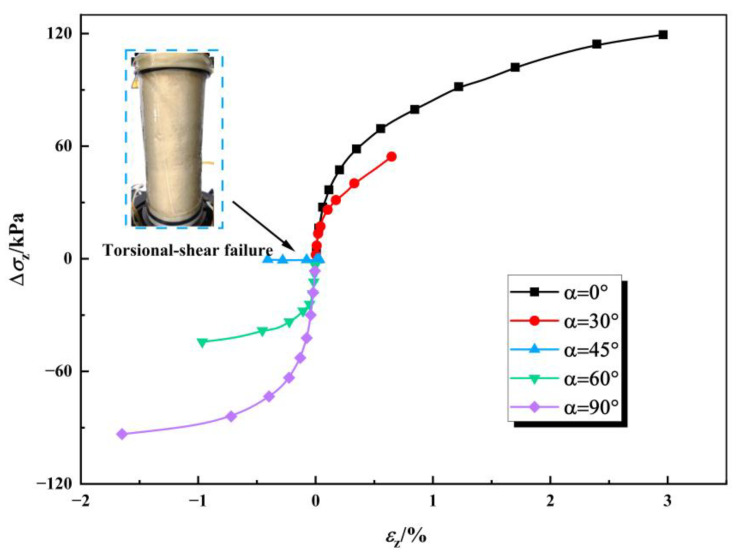
The relationship curve between axial stress variation and axial increment strain (*b* = 0.5, *c* = 5%).

Figure 9 illustrates the relationship curves between axial stress variation and axial strain increment at various deviation angles (*α*) of the principal stress axis. It is evident that the axial strain exhibits positive development when the deflection angle *α* ranges from 0° to 45°, while it shows negative development when *α* ranges from 45° to 90°. Furthermore, the axial strain increment progressively increases with the rise in axial stress. Within the deflection angle range of 0° to 45°, the positive amplitude of axial stress variation gradually diminishes. Conversely, as α increases from 45° to 90°, the negative amplitude of axial stress variation demonstrates a gradual increasing trend.

### 3.2. Stress–Strain Relationships Under Different Intermediate Principal Stress Coefficients

**Figure 10:**
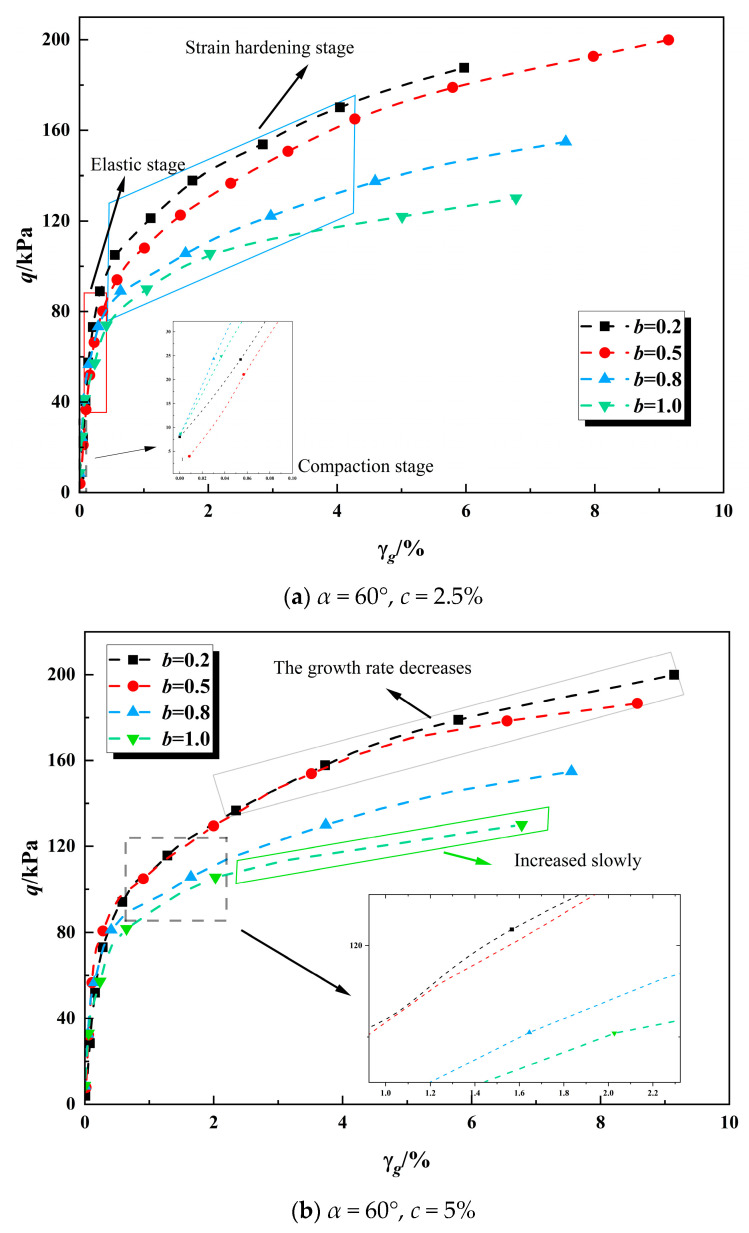

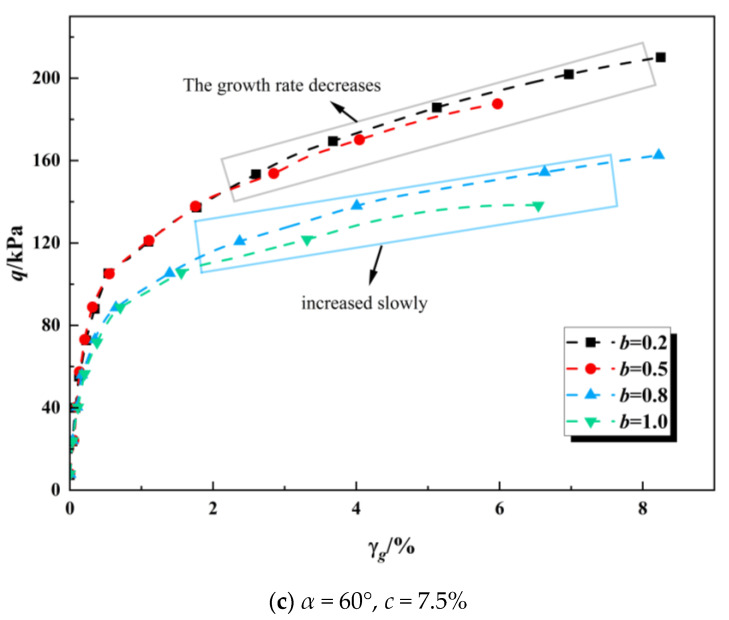
The relationship curve between deviatoric stress and generalized shear strain components under different values of *b*.

The experimental relationships between stress and strain components for hollow cylinder specimens at three polymer contents (*c* = 2.5%, 5% and 7.5%) are presented in Figure 10.

As illustrated in Figure 10, the deviatoric stress-generalized shear strain relationship of consolidated calcareous sand can be categorized into four distinct stages:

(1) Compaction Stage: In the initial loading phase, when the major principal strain is below 0.1%, the calcareous sand particles, the cementitious matrix of the consolidated calcareous sand, and the internal pores of the sample undergo rearrangement. This process transitions the sample towards a state capable of bearing load.
(2) Elastic Stage: The curve demonstrates a linear increase when the strain is within 0.5%, indicating the elastic nature of this stage. During this phase, the sample undergoes elastic deformation, which is fully recoverable upon unloading. Notably, the elastic modulus decreases as the intermediate principal stress coefficient increases.
(3) Strain Hardening Stage: In this phase, as the load progressively increases, the calcareous sand particles and cementitious materials within the sample begin to experience damage. The initiation of microcracks leads to a faster rate of strain increase relative to stress. This stage is critical for the onset of damage within the sample.
(4) Failure Stage: During this final stage, the stress exhibits minimal change while the strain increases rapidly, signifying that the sample has reached a state of failure.

Figure 10 also reveals that the maximum deviatoric stress of solidified calcareous sand increases, while the strain hardening trend diminishes, as the intermediate principal stress coefficient decreases. To further investigate the influence of polymer content on the specimen’s strength, subsequent sections of this paper will analyze the strength criteria of solidified calcareous sand in both the π-plane and the principal stress space.

### 3.3. Stress Component

**Figure 11:**
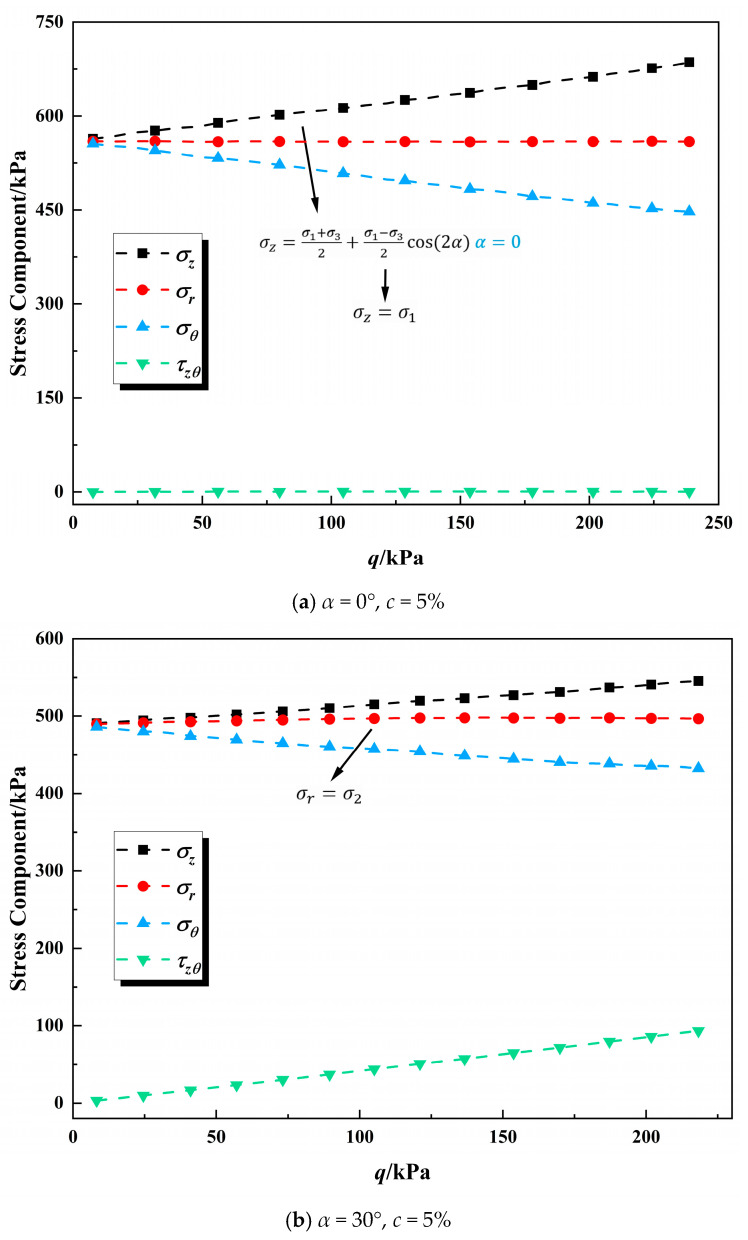

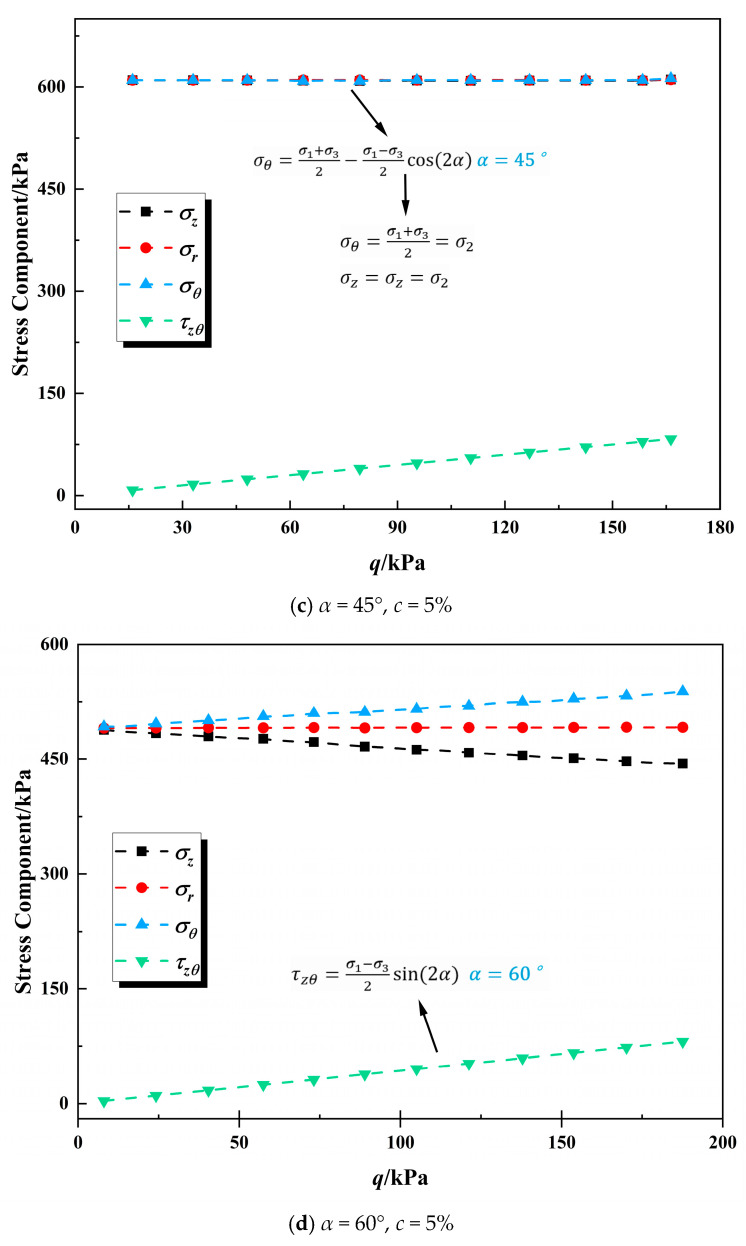

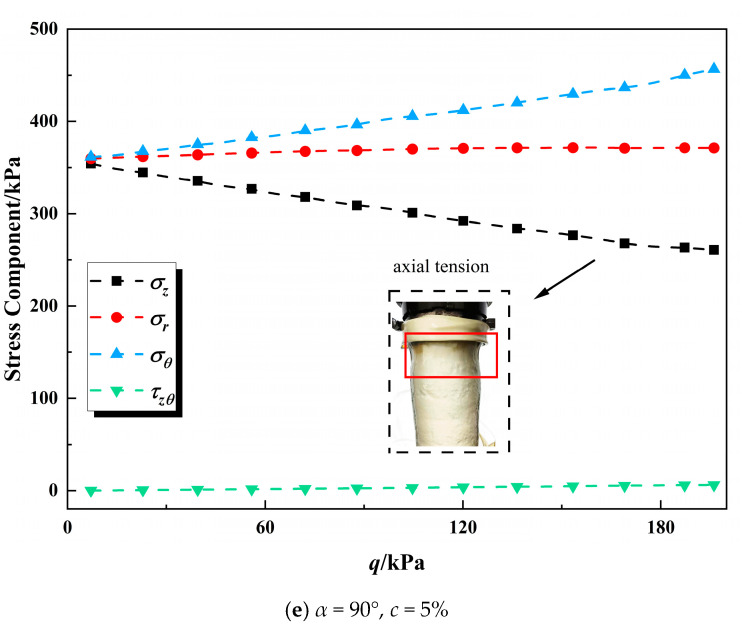
The relationship curves between stress components and deviatoric stress.

Figure 11 shows that during the process of fixed principal stress axes shearing the axial stress $\sigma_{z}$, radial stress ${\;\sigma }_{r}$, circumferential stress $\sigma_{\theta }$, and torsional shear stress $\tau_{z\theta }$ all maintain a relatively good linear relation with the generalized shear stress *q* regardless of the value of deflection angle *α*. This reflects the high level of stress control in all directions in this experiment, indicating that the test accuracy is reliable.

From Figure 11, the radial stress ($\sigma_{r}$) remains almost constant during the loading process. When the principal stress deflection angle *α* is within 0–45°, the axial stress ($\sigma_{z}$) gradually increases with *q*, but an increase in *α* contributes to a decrease in the growth rate of $\sigma_{z}$. The hoop stress ($\sigma_{\theta }$) decreases with the increase in deviatoric stress, and the decreasing amplitude decreases with the increase in the deviation angle of the principal stress; the torsional shear stress ($\tau_{z\theta }$) increases with the increase in deviatoric stress, and the increasing amplitude increases with the increase in the deviation angle of the principal stress. When the deviation angle of the principal stress is between 45° and 90°, the axial stress ($\sigma_{z}$) decreases with the increase in deviatoric stress, and the decreasing amplitude increases with the increase in the deviation angle of the principal stress; the hoop stress ($\sigma_{\theta }$) increases with the increase in deviatoric stress, and the increasing amplitude increases with the increase in the deviation angle of the principal stress; the torsional shear stress ($\tau_{z\theta }$) decreases with the increase in deviatoric stress, and the decreasing amplitude decreases with the increase in the deviation angle of the principal stress. As can be seen from the calculation formula in Table 3, the deviation angle of the principal stress is independent of radial stress and exists within the vertical and tangential planes, and is only related to vertical stress, tangential stress, and torsional shear stress. Combined with Figure 8, it can be concluded that when the sample is subjected to the coupled action of axial stress and torsional shear stress, the sample strength decreases significantly.

**Figure 12:**
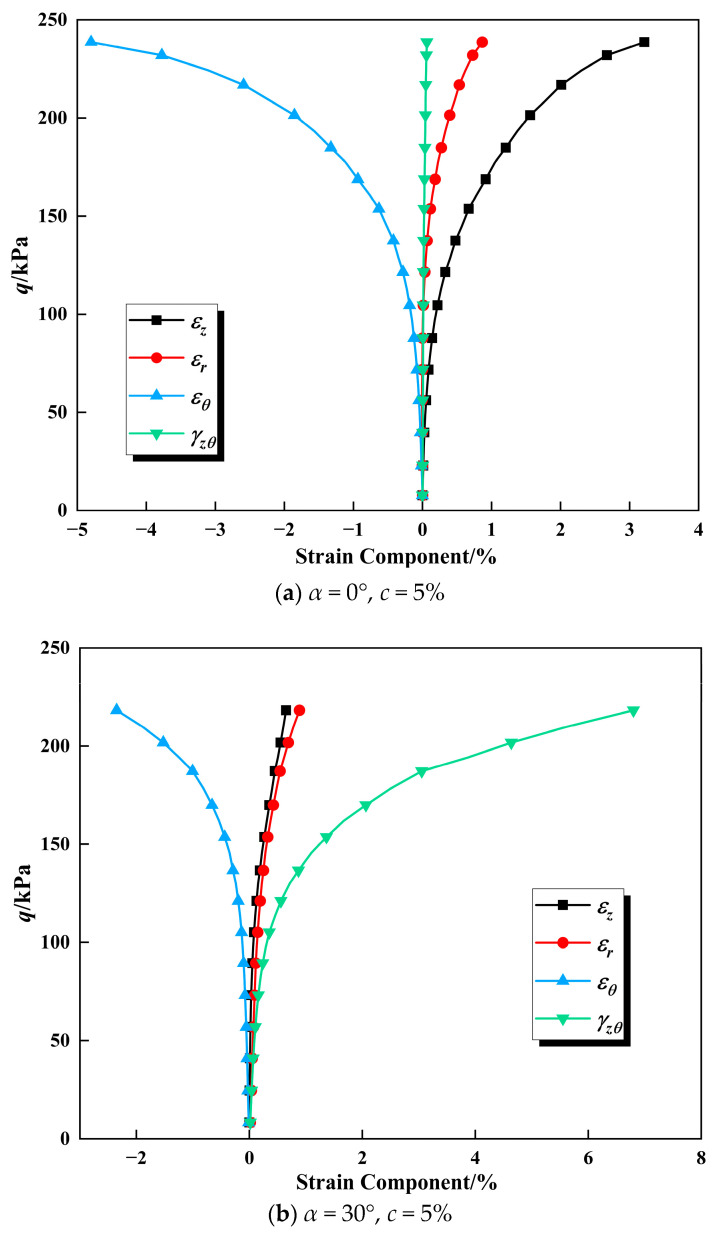

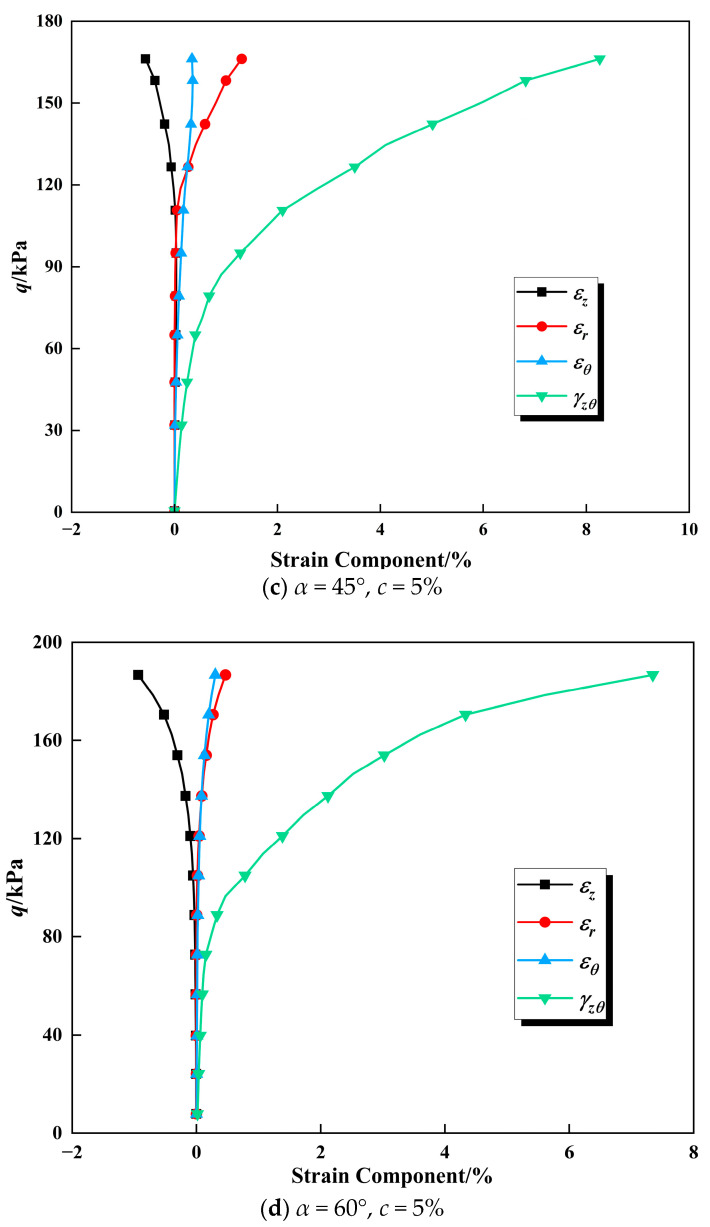

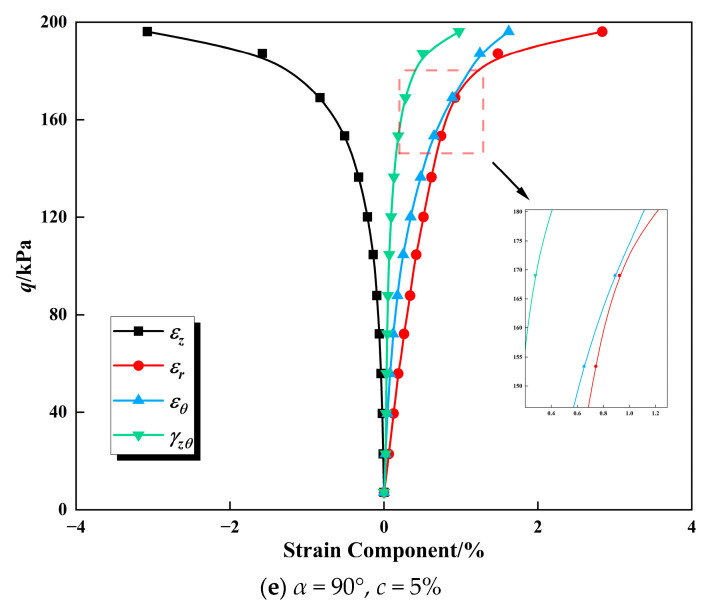
The relationship curve between strain components and deviatoric stress.

Figure 12 shows the relationship curves between generalized shear stress *q* and various strain components, including axial strain ($\varepsilon_{z}$), radial strain ($\varepsilon_{r}$), hoop strain ($\varepsilon_{\theta }$), and torsional shear strain ($\gamma_{z\theta }$). A typical nonlinear relationship can be observed between various strain components and *q*. With the increment of *q*, various strain components increase relatively slowly at the initial stage, and then rapidly increase. The changes in strain components in various directions are closely related to the stress state and the direction of the principal stress. When the direction angle (*α*) is 30°, the axial direction of the sample experiences a coupled effect of compression and torsional shear, resulting in a compressive state in the axial direction and an expansive state in the perpendicular hoop direction. Therefore, the axial strain ($\varepsilon_{z}$) manifests as positive strain, while the hoop strain ($\varepsilon_{\theta }$) is negative strain. Conversely, when the direction angle (*α*) is 60°, the axial direction of the sample experiences a coupled effect of tension and torsional shear, leading to opposite variations in the axial strain ($\varepsilon_{z}$) and hoop strain ($\varepsilon_{\theta }$). Within the five deflection angles studied in this paper, the torsional shear strain ($\gamma_{z\theta }$) consistently increased positively and had a larger value than the other strain components. Although the radial strain ($\varepsilon_{r}$) is theoretically zero, during the loading process, to achieve the stress path, various stress components need to be continuously adjusted, resulting in very small strain values. The differences in the variations in strain components at different major principal stress direction angles (*α*) are caused by the anisotropy of calcareous sand.

### 3.4. Non-Coaxial Characteristics

In the field of plasticity mechanics, non-coaxial characteristics are defined as the phenomenon where the direction of principal stress does not align with the direction of strain increment. Experimental evidence has confirmed that sandy soils exhibit non-coaxial characteristics under conditions of directional shearing and continuous rotation of the principal stress axis [44,45,46]. However, the specific manifestation of these non-coaxial characteristics varies depending on the type of sandy soil and the differences in loading paths.

**Figure 13:**
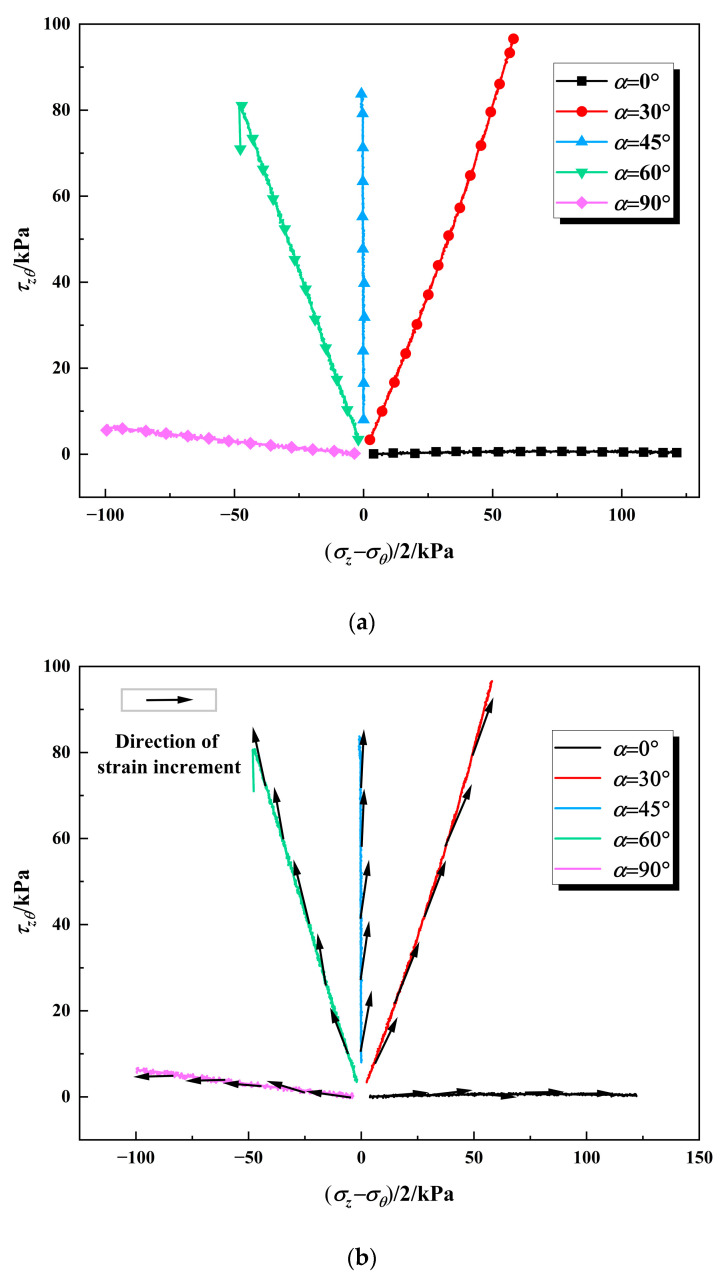
The schematic diagram of non-coaxial characteristics: (**a**) The direction of stress increment under different major principal stress angle; (**b**) The difference between strain increment and stress increment.

In the investigation of principal stress direction, the elastic strain constitutes a relatively small portion of the total strain, making its precise separation from the total strain challenging. Consequently, this study employs the total strain increment as a substitute to approximate the variation of plastic strain increment in the analysis. Utilizing the data presented in Table 4 and the experimental results, a schematic diagram illustrating the non-coaxial characteristics under various principal stress angles is derived, as depicted in Figure 13a.

As illustrated in Figure 13b, it is observed that when α is 0° or 90°, the strain increment direction of the solidified calcareous sand aligns with the stress direction, demonstrating a predominantly coaxial behavior. In general, the strain increment direction angle initially increases and then decreases as the generalized deviatoric stress increases, indicating that the degree of non-coaxiality first intensifies and subsequently diminishes. Specifically, for α values between 30° and 45°, the principal stress angle is smaller than the principal strain increment angle, leading to an increase in non-coaxial characteristics. Conversely, for α values between 45° and 60°, the principal stress angle is larger than the principal strain increment angle, resulting in a decrease in non-coaxial characteristics.

### 3.5. Strength Criterion of Polyurethane-Solidified Calcareous Sand

The strength criterion represents a fundamental research focus in island and reef engineering, as it defines the failure conditions of sandy soil masses and their capacity to resist external forces. The primary objective of strength theory research is to determine whether sandy soil materials fail under diverse and complex stress conditions. Over the past several decades, numerous scholars have proposed various strength criteria to characterize the failure behavior of sandy soils [29,30,31,35]. Nevertheless, these criteria exhibit limitations in accurately simulating the strength of solidified calcareous sand, particularly in capturing the variations in material strength associated with changes in polymer content. Consequently, there is a critical need to develop a strength criterion specifically for calcareous sand that incorporates the influence of polymer content. In this study, we employ a modified Lade criterion, which integrates the effect of polymer content, to effectively simulate the strength characteristics of the samples.

The Lade yield criterion was proposed in 1972 based on the true triaxial test to describe the initial yield condition of sandy soil under complex stress states. Its expression is related to the invariants of stress tensor. The Lade criterion is shown in Equation (5):

(5) $$I_{1}^{3}-{constant*I}_{3}=0$$

where

(6) $$I_{1}=\sigma_{1}+\sigma_{2}+\sigma_{3}$$

(7) $$I_{3}=\sigma_{1}\sigma_{2}\sigma_{3}$$

The *p*, *q* form of Lade criterion is as follows:

(8) $$q=\frac{6\sin\varphi }{3-\sin\varphi }g(\theta )p$$

where *g*(*θ*) is the shape function of the Lade criterion, and *φ* is the friction angle of sandy soil material.

The Lade criterion is characterized as a single-strength criterion with a fixed shape in the deviatoric plane, influenced solely by the material’s friction angle under varying Lode angles. However, it fails to adequately account for the effects of cohesion between materials or the interactions among different materials. Given that the cohesion of polymer-solidified calcareous sand is significant and cannot be overlooked, the Lade criterion has been modified to develop the Unified Linear Strength (ULS) criterion [47]. The expression for this modified criterion is as follows:

(9) $$q=\frac{6\sin\varphi }{3-\sin\varphi }g_{u}\left(\begin{array}{c} \theta \end{array}\right)\left(\begin{array}{c} p+\sigma_{0} \end{array}\right)$$

where $\sigma_{0}\;$ is the spherical stress, which reflects the cohesion characteristics of the material and can be obtained based on the intercept and slope of the *p*-*q* plane; $g_{u}\left(\begin{array}{c} \theta \end{array}\right)$ is the shape function of the Unified Linear Strength criterion.

(10) $$g_{u}\left(\begin{array}{c} \theta \end{array}\right)=\frac{r_{\theta }}{r_{0}}$$

where

(11) $$r_{\theta }=\frac{\sqrt{3}}{2\sqrt{A_{1}}\cos\delta_{\theta }}+\frac{6\xi \sin\varphi }{3-sin\varphi }$$

(12) $$r_{0}=\frac{\sqrt{3}}{2\sqrt{A_{1}}\cos\delta_{0}}+\frac{6\xi \sin\varphi }{3-\sin\varphi }$$

where $A_{1}\;$ is a constant variable related to the friction angle of the material, and its specific calculation formula is as follows:

(13) $$A_{1}=\frac{{\left(3-\sin\varphi \right)}^{3}}{3{\left(3-\sin\varphi \right)}^{3}-81\left(1-\sin\varphi -\sin^{2}\varphi +\sin^{3}\varphi \right)}$$

where *ξ* is the model parameter. $\delta_{\theta }\;$ and $\delta_{0}$ can be expressed as follows:

(14) $$\delta_{\theta }=\frac{1}{3}arccos\left(-\frac{3\sqrt{3}A_{3}\cos\left(3\theta \right)}{2A_{2}^{\frac{3}{2}}}\right)$$

(15) $$\delta_{0}=\frac{1}{3}arccos\left(-\frac{3\sqrt{3}A_{3}}{2A_{2}^{\frac{3}{2}}}\right)$$

where

(16) $$A_{2}=\frac{{\left(3-sin\varphi_{0}\right)}^{3}}{3{\left(3-sin\varphi_{0}\right)}^{3}-81\left(1-sin\varphi_{0}-{sin}^{2}\varphi_{0}+{sin}^{3}\varphi_{0}\right)}$$

(17) $$A_{3}=\frac{2{\left(3-sin\varphi_{0}\right)}^{3}}{27{\left(3-sin\varphi_{0}\right)}^{3}-729\left(1-sin\varphi_{0}-{sin}^{2}\varphi_{0}+{sin}^{3}\varphi_{0}\right)}$$

**Figure 14:**
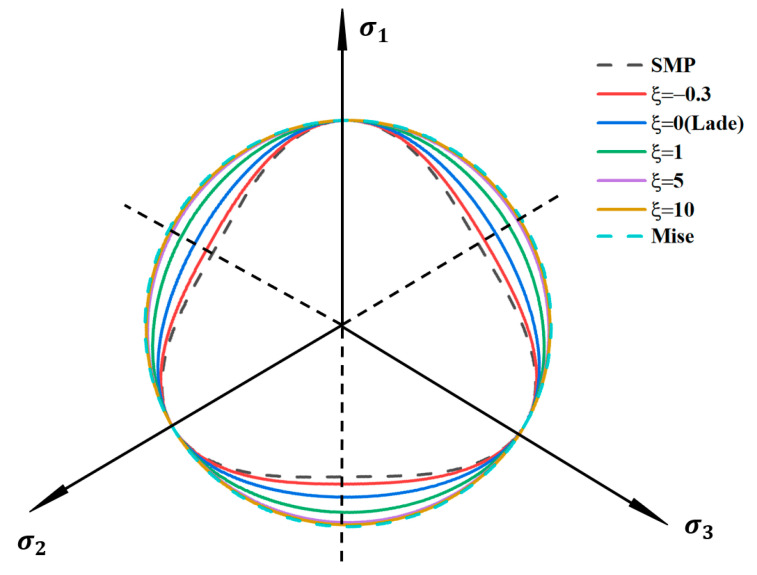
Envelope curves under different values of *ξ*.

When the model parameter *ξ* gradually increases from −0.3 to 10, the shape of the criterion transitions from the SMP curved-side triangle to the Mises circle. The modified linear strength criterion is a series of smooth curves in the π-plane. With other parameters fixed and only the model parameter *ξ* changed, the projection of this criterion in the deviatoric plane is shown in Figure 14.

We have added a strength parameter *ω* that can reflect the polymer content on the basis of the Unified Linear Strength criterion.

(18) $$q=\frac{6\omega \sin\varphi }{6-2\sin\varphi }g_{u}\left(\begin{array}{c} \theta \end{array}\right)\left(\begin{array}{c} p+\sigma_{0} \end{array}\right)$$

where

(19) $$\omega =-1.2c^{2}+19c+105$$

As indicated by Equation (19), the strength of the solidified sample exhibits an initial increase with polymer content within a specific range, albeit at a progressively diminishing rate. Beyond this range, however, further increases in polymer content lead to a reduction in sample strength. The peak strength is achieved at a polymer content of *c* = 8%.

**Figure 15:**
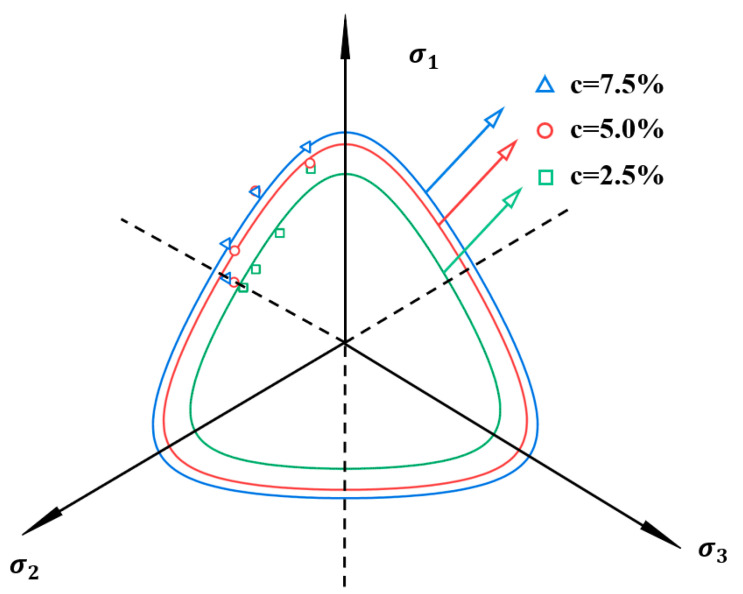
Prediction of envelope curves in the *π*-plane.

As illustrated in Figure 15, the experimental failure strength of the sample demonstrates excellent agreement with the theoretical predictions. The proposed model effectively captures the continuous stress redistribution within the boundary surface, thereby maintaining the material in a plastic loading state. Within a specific range, the failure strength exhibits a gradual increase with the increment of polymer content (c). According to Dong et al. [48], in transversely isotropic materials, there exists a set of potential sliding surfaces characterized by a constant ratio of shear stress to normal stress on each surface. However, the material strength varies across different potential sliding surfaces. Consequently, it can be inferred that sample failure consistently occurs along the potential sliding surface with the minimum strength, where sliding tendency is most pronounced. The peak strength of polymer-stabilized calcareous sand is consequently governed by the weakest potential sliding surface. Higher polymer content contributes to enhanced stability of the failure surface, resulting in superior material strength.

Based on the strength curve in the *p-q* plane and the shape function of the modified Lade criterion in the *π*-plane, an intensity criterion for solidified calcareous sand in the three-dimensional principal stress space is proposed.

(20) $$J_{2}=g_{u}\left(\begin{array}{c} \theta \end{array}\right)\cdot f\left(I_{1}\right)$$

where $J_{2}$ represents the second stress invariant, and the meridian plane $f\left(I_{1}\right)$ is a function of $I_{1}$, with the specific formula as follows:

(21) $$f\left(I_{1}\right)=\frac{C\cdot I_{1}}{2\sqrt{3}}$$

**Figure 16:**
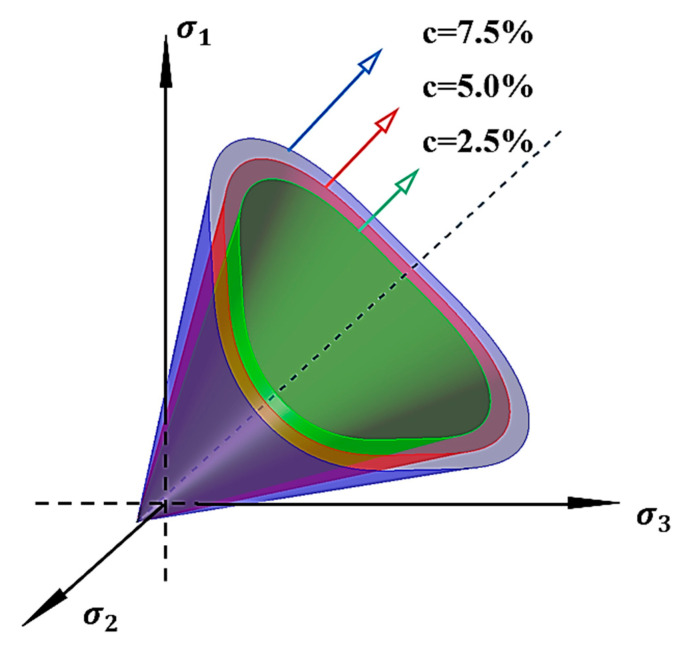
The failure envelope in the three-dimensional principal stress space.

Based on the equivalent model established in this study, the calculated model parameters were programmed to determine the yield surface shape of the modified Lade criterion in the stress space, as illustrated in Figure 16. The failure surface of solidified calcareous sand exhibits the following characteristics: (1) In the principal stress space, its shape is a smooth triangular cone. (2) Owing to the tensile strength present in solidified calcareous sand, the failure surface does not intersect the origin but instead passes through the isotropic tensile point.

## 4. Conclusions

A series of hollow cylinder tests were conducted to investigate the deformation and strength behavior of polyurethane (PFA)-cemented calcareous sand under complex stress states, systematically considering polymer content (*c* = 2.5%, 5%, and 7.5%), major principal stress rotation angle (α = 0°, 30°, 45°, 60°, and 90°), and Lode angle ($\theta_{\sigma }$ = −19.1°, 0°, 19.1°, and 30°). A novel three-dimensional strength criterion for solidified calcareous sand was developed and validated based on experimental findings. Key conclusions are summarized as follows:

(1) Shear Deformation Behavior and Quantitative Characterization

The shear deformation mechanisms of PFA-cemented calcareous sand under complex stress states were investigated using hollow cylinder tests. The material exhibited pronounced strain hardening during fixed major principal stress axis shearing, with the generalized shear strain growth rate decelerating beyond 6%. Consequently, the deviatoric stress at 6.5% strain (deviation < 5%) was defined as the failure strength. As the major principal stress direction angle α increased from 0° to 45°, the failure strength declined by 30% (at PFA content *c* = 5%), while further rotation to α = 90° triggered a 25% strength recovery. This reveals an anisotropic regulatory mechanism governing deformation via principal stress axis rotation. Non-coaxial strain–stress behavior (maximum deviation angle: 15°) further corroborates the critical role of internal particle–polymer interactions in deformation evolution.

(2) Multiaxial Strength Behavior and Criterion Development

The multiaxial strength of PFA-cemented calcareous sand is governed by the coupled effects of mean principal stress (*p*), principal stress rotation angle (*α*), and stress Lode angle ($\theta_{\sigma }$). In the *p*-*q* plane, linear strength envelopes were observed, with their slopes increasing markedly with PFA content (e.g., a 15% enhancement at *c* = 7.5% compared to *c* = 2.5%). The π-plane strength envelope adopts a curved triangular form, accurately captured by a modified Lade criterion incorporating PFA content as a key parameter (prediction error < 10%). This criterion quantifies $\theta_{\sigma }$-dependent strength modulation, demonstrating a 20% strength increase under $\theta_{\sigma }$ = −19.1° (compression-dominant) versus $\theta_{\sigma }$ = 30° (tension-dominant) conditions.

(3) Formulation and Validation of 3D Strength Criterion

A unified 3D strength criterion was established by coupling the *p*-*q* plane linearity with the π-plane modified Lade function. The resultant failure surface in principal stress space is continuous and smooth, comprehensively characterizing the synergistic effects of PFA content (2.5–7.5%), principal stress orientation (α = 0–90°), and stress state ($\theta_{\sigma }$ = −19.1–30°) on material strength. This framework provides a robust theoretical tool for analyzing calcareous sand engineering responses under complex stress paths.

## Figures and Tables

**Table 1:** Summary of key features of the SS-HCA.

Type of Measurement	Capacity	Maximum Error or Accuracy *
Axial load	−20~20 kN	0.1%
Axial displacement	−40~40 mm	0.001 mm
Torque	−400~400 N·m	0.1%
Torque displacement	−114~114 mm	0.001 mm
Torsional angle	−45°~45°	0.1%
Internal and outer cell pressure	0~2 MPa	0.25%

* % errors are based on full-scale output.

**Table 2:** Summary of directional shear tests.

Test Nos.	*c* (%)	α (°)	*p*′ (kPa)	*b*
T1	5%	0	100	0.5
T2	5%	30	100	0.5
T3	5%	45	100	0.5
T4	5%	60	100	0.5
T5	5%	90	100	0.5
T6–T9	2.5%	60	100	0.2, 0.5, 0.8, 1.0
T10–T12	5%	60	100	0.2, 0.8, 1.0
T12–T16	7.5%	60	100	0.2, 0.5, 0.8, 1.0

**Table 3:** Equations for data interpretation.

	Stress Components	Strain Components
Axial	$\sigma_{z}=\frac{W}{\pi (r_{o}^{2}-r_{i}^{2})}+\frac{p_{o}r_{o}^{2}-p_{i}r_{i}^{2}}{\left(r_{o}^{2}-r_{i}^{2}\right)}$	$\varepsilon_{z}=\frac{\omega }{H}$
Radial	$\sigma_{r}=\frac{p_{o}r_{o}+p_{i}r_{i}}{r_{o}+r_{i}}$	$\varepsilon_{r}=-\frac{u_{o}-u_{i}}{r_{o}-r_{i}}$
Circumferential	$\sigma_{\theta }=\frac{p_{o}r_{o}-p_{i}r_{i}}{r_{o}-r_{i}}$	$\varepsilon_{\theta }=-\frac{u_{o}+u_{i}}{r_{o}+r_{i}}$
Torsional shear	$\tau_{z\theta }=\frac{3M_{T}}{2\pi (r_{o}^{3}-r_{i}^{3})}$	$\gamma_{z\theta }=\frac{2\theta \left(r_{o}^{3}-r_{i}^{3}\right)}{3H\left(r_{o}^{2}-r_{i}^{2}\right)}$
Major principal	$\sigma_{1}=\frac{\sigma_{z}+\sigma_{\theta }}{2}+\sqrt{{\left(\frac{\sigma_{z}-\sigma_{\theta }}{2}\right)}^{2}+{\tau_{z\theta }}^{2}}$	$\varepsilon_{1}=\frac{\varepsilon_{z}+\varepsilon_{\theta }}{2}+\sqrt{{\left(\frac{\varepsilon_{z}-\varepsilon_{\theta }}{2}\right)}^{2}+\gamma_{z\theta }^{2}}$
Intermediate principal	$\sigma_{2}=\sigma_{r}$	$\varepsilon_{2}=\varepsilon_{r}$
Minor principal	$\sigma_{3}=\frac{\sigma_{z}+\sigma_{\theta }}{2}-\sqrt{{\left(\frac{\sigma_{z}-\sigma_{\theta }}{2}\right)}^{2}+{\tau_{z\theta }}^{2}}$	$\varepsilon_{1}=\frac{\varepsilon_{z}+\varepsilon_{\theta }}{2}-\sqrt{{\left(\frac{\varepsilon_{z}-\varepsilon_{\theta }}{2}\right)}^{2}+\gamma_{z\theta }^{2}}$

**Table 4:** The formula for calculating non-coaxial characteristics.

Principal stress orientation angle	$\alpha =\frac{1}{2}arctan\frac{2\tau_{z\theta }}{\sigma_{z-}\sigma_{\theta }}$
Direction angle of principal strain rate	$\alpha_{d\varepsilon }=\frac{1}{2}arctan\frac{2d\varepsilon_{z\theta }}{d\varepsilon_{z}-d\varepsilon_{\theta }}$
Non-coaxial angle	$\beta =\alpha_{d\varepsilon }-\alpha$
Stress increment	$ds=\sqrt{{\left[d\left(\frac{\sigma_{z}-\sigma_{\theta }}{\sigma_{z}+\sigma_{\theta }}\right)\right]}^{2}+{\left[d\left(\frac{2\tau_{z\theta }}{\sigma_{z}+\sigma_{\theta }}\right)\right]}^{2}}$
Strain increment generated by unit stress	$\varepsilon_{s}=\frac{\sqrt{{\left[d\left(\varepsilon_{z}-\varepsilon_{\theta }\right)\right]}^{2}+{\left[d\gamma_{z\theta }\right]}^{2}}}{ds}=\frac{d\varepsilon_{1}-d\varepsilon_{3}}{ds}$

## Data Availability

Raw data that support the finding of this study are available from the corresponding author upon request.
